# Spatial-temporal mapping and risk factors for hand foot and mouth disease in northwestern inland China

**DOI:** 10.1371/journal.pntd.0009210

**Published:** 2021-03-24

**Authors:** Ruifang Huang, Jiate Wei, Zhenwei Li, Zhenguo Gao, Muti Mahe, Wuchun Cao

**Affiliations:** 1 Xinjiang Uighur Autonomous Region Center for Disease Control and Prevention, Urumqi, P. R. China; 2 Institute of EcoHealth, School of Public Health, Cheeloo College of Medicine, Shandong University, Jinan, P. R. China; 3 State Key Laboratory of Pathogen and Biosecurity, Beijing Institute of Microbiology and Epidemiology, Beijing, P. R. China; Institute for Disease Modeling, UNITED STATES

## Abstract

**Background:**

Hand foot and mouth disease (HFMD) is becoming one of the common human infectious diseases in China. Previous studies have described HFMD in tropical or coastal areas of Asia-Pacific countries. However, limited studies have thoroughly studied the epidemiology and potential risk factors for HFMD in inland areas with complex environmental conditions.

**Methodology/Principal findings:**

Using the data from 2009 to 2018 on reported cases of Xinjiang Uighur Autonomous Region, we characterized the epidemic features of HFMD. Panel negative binomial model was used to identify climate, geographical and demographic determinants for HFMD incidence. A total of 70856 HFMD cases (average annual incidence: 305 per million persons) were reported in Xinjiang during the 10-year study period, of which 10393 (14.7%) were laboratory-confirmed and 98 (0.1%) were severe. HFMD peaked in summer every year during the study period, and incidence in 2012, 2015, 2016 and 2018 had minor peaks in autumn. After adjusting the school or holiday month, multiple factors were found to affect HFMD epidemiology: urban area being major land cover type (incidence risk ratio, IRR 2.08; 95% CI 1.50, 2.89), higher gross domestic product per capita (IRR 1.14; 95% CI 1.11, 1.16), rise in monthly average temperature (IRR 1.65; 95% CI 1.61, 1.69) and monthly accumulative precipitation (IRR 1.20; 95% CI 1.16, 1.24) predicted increase in the incidence of HFMD; farmland being major land cover type (IRR 0.72; 95% CI 0.64, 0.81), an increase of percentage of the minority (IRR 0.91; 95% CI 0.89, 0.93) and population density (IRR 0.98; 95% CI 0.98, 0.99) were related to a decrease in the incidence of HFMD.

**Conclusions/Significance:**

In conclusion, the epidemic status of HFMD in Xinjiang is characterized by low morbidity and fatality. Multiple factors have significant influences on the occurrence and transmission of HFMD in Xinjiang.

## Author summary

Hand foot and mouth disease (HFMD) is one of the common human infectious disease threating Asia-Pacific countries. To explore the epidemiology and environmental risk factors for HFMD in inland China, we utilized 10-year HFMD surveillance data in Xinjiang Uighur Autonomous Region and combined multiple spatial-temporal statistical analyses. We identified spatial-temporal clusters of HFMD incidence and found that multiple factors could affect HFMD incidence: urban area being major land cover type, higher gross domestic product per capita, rise in monthly average temperature and monthly accumulative precipitation predicted increase in the incidence of HFMD; farmland being major land cover type, an increase of percentage of the minority and population density were related to a decrease in the incidence of HFMD. Our findings facilitate the understanding of HFMD epidemiology and risk factors in different geographic regions, which are crucial for conducting prevention and control strategies of HFMD.

## Introduction

Hand foot and mouth disease (HFMD) is a common viral infectious disease, which is mainly caused by *coxsackie A16* (CV-A16), *enterovirus 71* (EV71) and other enteroviruses. In most cases, the symptoms of HFMD are mild and self-limiting, including fever, papular skin on hand and feet, or vesicular rash in the mouth. However, severe neurological and systemic complications that may be fatal can also develop rapidly, especially in children younger than 5 years. In past decades, HFMD has become one of the major public health issues in Asia-Pacific countries, attracting attention from researchers worldwide [1]. Particularly, the disease burden of HFMD in China is very heavy, with 1.2 per 1000 person-years of incidence and 500–900 deaths every year in 2010–2012 [2]. In May 2008, the Chinese Ministry of Health listed HFMD as class “C” notifiable disease and started the disease surveillance in the whole country.

The spatial and temporal pattern of HFMD incidence has been reported by various studies in Asia-Pacific regions like Singapore, Malaysia, Japan, South Korea, Taiwan, Hong Kong, and mainland China [1–9]. The peaks of HFMD were observed to occur in different months from spring to autumn, and the periodicities were found to be annual, semi-annual or insignificant in the above areas. Many climate and demographic factors were also reported to be associated with HFMD incidence, yet the significance and direction of correlations have been quite inconsistent [3,6,7,10–16]. Most studies on climate factors found the positive association between temperature and HFMD incidence, but conflicting relationships were reported of HFMD incidence with relative humidity, precipitation, atmospheric pressure, wind speed and sunshine duration [3,6,7,10–13]. Inconsistent findings on HFMD incidence and demographic factors such as gross domestic product (GDP) and population density were also reported [10,11,14,15]. These divergences may due to changes in the study area and model fitting. It should be noted that most previous studies on potential factors for HFMD laid the focus in east or south Asian regions near the ocean, limited studies have focused in inland regions [9–11]. Considering the relative drier climate and more complex environmental conditions of the inland area (the interior area of the country), the epidemiology of HFMD in the inland area may be different and are needed to be thoroughly studied. Therefore, the present study aims to investigate the epidemiology, viral etiology, and potential effects of climate, geographical and demographic factors of HFMD through 2009–2018 in Xinjiang Uighur Autonomous Region, northwestern inland China.

## Materials and methods

### Ethics statement

As this study constituted data analysis rather than research in human beings, ethical approval from institutional review boards was not required.

### Study area

Xinjiang Uighur Autonomous Region is the biggest provincial-level administrative region in China and spans over 1.66 million km^2^. Xinjiang is situated in around latitude 34°22′ to 49°10′ N and longitude 73°40′ to 96°23′ E, with over 24 million registered residents in 2018 (S1 Fig). The climate of Xinjiang is generally characterized as temperate continental climate but varies largely among different regions due to the complicated natural conditions (farms, deserts, grasslands, mountains, et al.). The ethnic-minority proportion in Xinjiang is very high compared to other provincial administrative region in China, including Uighur, Kazak, Hui, Kirgiz, Mongolian and other minorities.

### HFMD surveillance data

Data on daily HFMD cases from January 2009 to December 2018 were obtained from Xinjiang Center for Disease Control and Prevention (CDC). Within 24 hours after diagnosis, the HFMD cases were mandatorily reported online. The individual information of patients was collected, including basic demographics (sex, age, county of residence); case classification (probable or confirmed); disease severity (severe or mild); date of onset, diagnosis, and death (if applicable); and enterovirus serotype (for confirmed cases). The HFMD case was defined as follows: probable case who had papular or vesiculation on hands, feet, buttocks or in the mouth, with or without fever; and confirmed case who is the probable patient with laboratory evidence of enterovirus infection (EV71, CV-A16 and other enterovirus).

### Climate, geographical, and demographic data

To analyze the potential drivers of HFMD, we collected climate, geographical, and demographic information in Xinjiang from 2009 to 2018. Base maps were downloaded from National Earth System Science Data Center, National Science & Technology Infrastructure of China (http://www.geodata.cn). Climate data of all 66 stations in Xinjiang were downloaded from the National Meteorological Information Center (http://data.cma.cn/data/cdcindex.html). Elevation data with ~1km resolution were derived from the Shuttle Radar Topography Mission (SRTM) (http://www.resdc.cn/data.aspx?DATAID=123). Land cover data at a resolution of ~1km were extracted from the Landsat 8 remote sensing map (http://www.resdc.cn/data.aspx?DATAID=184). In each county, monthly mean temperature, relative humidity, atmospheric pressure, sunshine duration and accumulative precipitation were extracted by Kriging interpolation and zonal statistics in ArcGIS 10.2 (ESRI, ArcGIS 10.2, Redlands, CA, USA); mean elevation and major type of land cover (farmland, forest, grassland, water body, urban area and unused land) were also extracted by zonal statistics. For demographic data, annual population size, percentage of the minority and GDP per capita by county from 2009 to 2018 were extracted from Xinjiang Statistical Yearbook. The annual population density of each county was obtained by dividing population size by the land area.

### Data analysis

All HFMD cases with illness onset from Jan 1, 2009 to Dec 31, 2018 were included in our analysis. The county-level administrative divisions in Xinjiang varied from 2009 to 2018, and we had the map of Xinjiang in version 2013 for visualization. To unify the statistical scale, cases and demographic variables in counties assigned after 2013 was included in 101 county-level administrative regions in 2013. We summarized the demographic characteristics of cases and compared them between the two groups of sex. The age-specific annual incidence rate was calculated by combining probable and confirmed cases. The 95% confidence intervals (CIs) were estimated with Poisson methods. Serotype-specific analyses were conducted for confirmed cases to estimate the serotype distributions of enterovirus in different years and age groups. To identify the periodicity of HFMD in Xinjiang, Continuous Wavelet Transform (CWT) was conducted in “WaveletComp” R package. Geographical and temporal distribution of cases were assessed across all 101 counties of Xinjiang, and spatial-temporal clusters from 2009 to 2018 were found using SaTScan 9.6 (www.satscan.org) with discrete Poisson model.

Then, we explored the potential effects of climate, geographical and demographic factors on reported HFMD incidence. The explanatory variables were extracted in specific time scale by county: monthly mean temperature (°C), relative humidity (%), atmospheric pressure (Hpa) and sunshine duration (0.1h), monthly accumulative precipitation (mm); mean elevation (m), major type of land cover (unordered categorical: farmland, forest, grassland, water body, urban area and unused land); the yearly percentage of the minority (%), GDP per capita (10 000 yuan) and population density (per /km^2^). The dependent variable was monthly count of cases in each county. Panel negative binomial regression was conducted with offset for the population. In this model, the time trend and correlation between the observations were adjusted by identifying the panel group (individual county) and time index (by month) of the data. This model also generically accounted for the overdispersion caused by the clustered count data [17,18]. To control the potential confounds caused by schooling of students, January, February, July and August were classified as holiday months according to the Chinese system of winter and summer holidays arrangement. Other months in a year were defined as school month. School or holiday month was included into both the univariate model and multivariate models as adjustment. Univariate analysis was conducted at first for each covariate, variables for which *p*<0.1 were included in the multivariate model. To deal with the collinearity between variables, Spearman correlation tests were conducted including all continuous variables. For highly correlated variables (coefficient>0.7), a single variable was selected for inclusion on the basis of marginal Akaike Information Criterion (AIC). Variance inflation factors (VIFs) were also calculated, and explanatory variables with VIF>10 were removed from the model (S1 Text). The above regression models were conducted in Stata 15.1.

## Results

During 2009–2018, a total of 70856 HFMD cases were reported in Xinjiang, of which 98 (0.1%) were severe and 11 (0.02%) were fatal. In the cases, 42475 (59.9%) were male and 28381 (40.1%) were female, leading 1.45-times-higher incidence rate in male. The median age of HFMD onset is 3.0 (IQR 2.0–4.0), and a significant difference was found in age distributions between male and female (*p*<0.001). Higher proportion of the cases younger than 5 years was found in male than in female. No statistical difference was seen in the distribution of virus subtypes between male and female (Table 1). During 2009–2018, the incidence rate of HFMD varied largely among age groups, with the highest rates in children aged 1–5 years. The incidences of HFMD were very low in people under one year and over 5 years old (Table 2).

**Table 1:** Total and sex-stratified characteristics of HFMD cases.

	Total	Female	Male	*p*
No. patients	70856	28381	42475	
Severe illness (%)	98 (0.1)	38 (0.1)	60 (0.1)	0.876
Age (median (IQR))	3.0 (2.0, 4.0)	3.0 (2.0, 4.0)	3.0 (1.0, 4.0)	<0.001*
Age groups (%)				<0.001*
0-12months	3390 (4.8)	1265 (4.5)	2125 (5.0)	
1 year	14258 (20.1)	5668 (20.0)	8590 (20.2)	
2–4 years	37928 (53.5)	14967 (52.7)	22961 (54.1)	
5–9 years	12660 (17.9)	5206 (18.3)	7454 (17.5)	
10–14 years	1600 (2.3)	711 (2.5)	889 (2.1)	
≥15 years	1020 (1.4)	564 (2.0)	456 (1.1)	
Etiological detection results (%)				0.331
Probable	60463 (85.3)	24276 (85.5)	36187 (85.2)	
Other enterovirus	3442 (4.9)	1346 (4.6)	2096 (4.7)	
EV71	2864 (4.0)	1112 (3.9)	1752 (4.1)	
CV-A16	4087 (5.8)	1647 (5.8)	2440 (5.5)	

* *P* is statistically significant (*p* < 0.05).

**Table 2:** Estimated incidence rate and 95% confidence interval of HFMD by year and age group (per million persons)*.

Age	2009	2010	2011	2012	2013	2014	2015	2016	2017	2018
Median (IQR)	3 (1, 4)	3 (2, 4)	3 (2, 4)	3 (2, 4)	3 (1, 4)	3 (2, 4)	3 (1, 4)	3 (1, 4)	3 (1, 4)	3 (2, 5)
0-12months	668.5 (617.9, 719.2)	594.5 (546.7, 642.3)	534.8 (489.5, 580.2)	783.1 (728.3, 838)	687.6 (636.2, 739)	625.6 (576.5, 674.6)	924 (864.4, 983.6)	1616.4 (1537.6, 1695.2)	491.9 (448.4, 535.3)	1167.6 (1100.6, 1234.5)
1 year	3057.1 (2948.7, 3165.5)	2863.2 (2758.3, 2968.1)	2305.2 (2211.1, 2399.3)	3641.1 (3522.8, 3759.4)	2707.2 (2605.2, 2809.2)	3054.7 (2946.4, 3163.1)	3794.8 (3674, 3915.5)	5799.7 (5650.5, 5949)	1685.8 (1605.3, 1766.3)	4802 (4666.2, 4937.8)
2–4 years	2923.5 (2817.5, 3029.5)	3395.4 (3281.2, 3509.6)	2827.2 (2723, 2931.4)	4286.3 (4158, 4414.6)	2475.9 (2378.3, 2573.4)	3368.5 (3254.8, 3482.3)	3222.8 (3111.5, 3334)	4299.3 (4170.8, 4427.9)	1607.5 (1528.9, 1686.1)	4496.3 (4364.8, 4627.7)
5–9 years	388.2 (349.6, 426.8)	520.6 (475.9, 565.3)	449.9 (408.3, 491.4)	791.9 (736.8, 847.1)	495 (451.4, 538.6)	663.5 (613, 714)	603.3 (555.2, 651.5)	920.3 (860.8, 979.8)	317 (282.1, 351.9)	1199.7 (1131.8, 1267.5)
10–14 years	85.9 (67.8, 104.1)	85.3 (67.2, 103.4)	51.7 (37.6, 65.8)	109.6 (89.1, 130.1)	109 (88.5, 129.4)	97.2 (77.8, 116.5)	105.9 (85.7, 126)	140.1 (116.9, 163.3)	56.7 (41.9, 71.4)	155.1 (130.7, 179.5)
≥15 years	3.3 (-0.3, 6.8)	3.9 (0, 7.7)	2.2 (-0.7, 5.2)	6 (1.2, 10.8)	5 (0.6, 9.4)	4.2 (0.2, 8.2)	6.2 (1.3, 11.1)	10 (3.8, 16.2)	2.9 (-0.4, 6.3)	10.4 (4.1, 16.7)

* 95% confidence intervals were estimated with Poisson methods.

**Fig 1:**
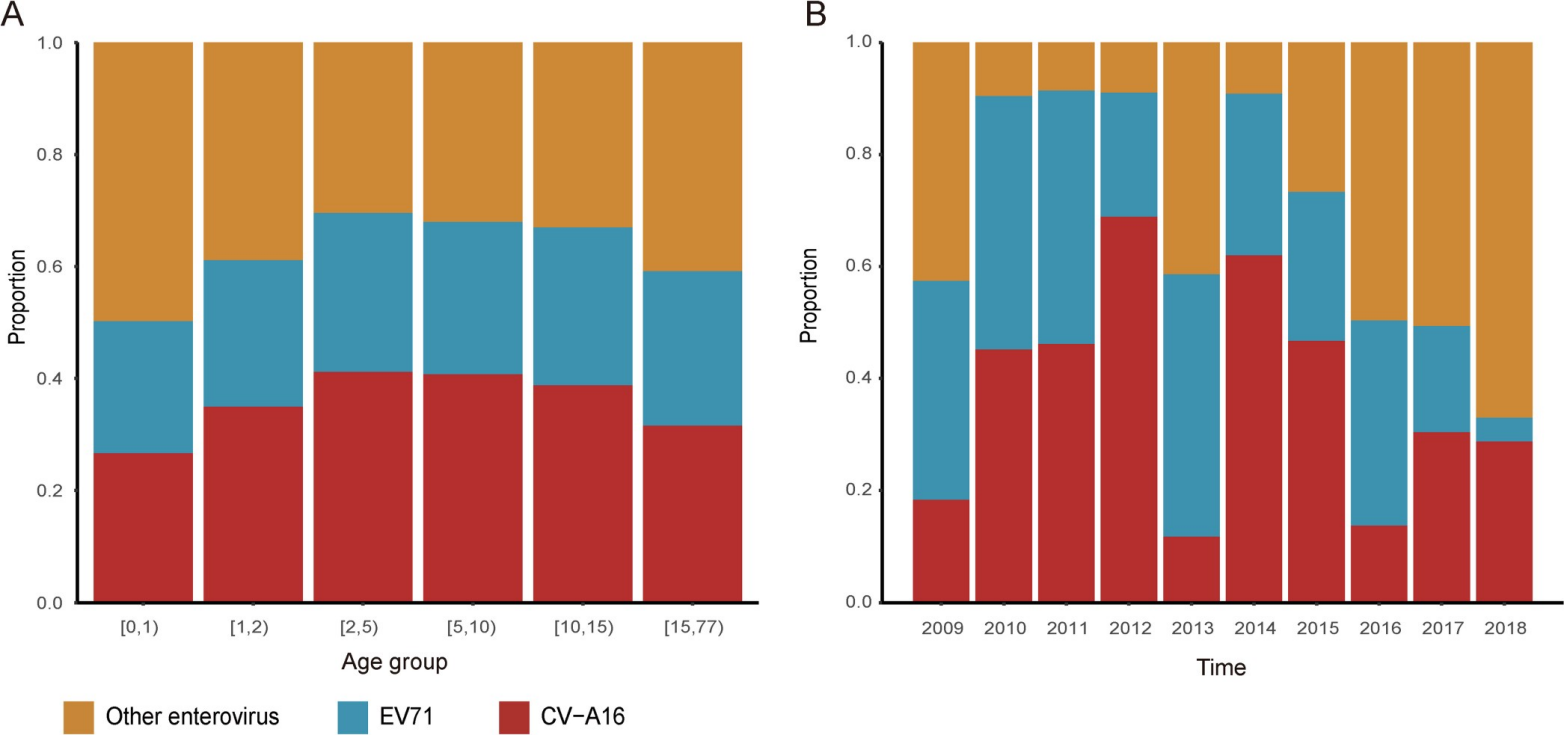
Virus etiology of hand foot and mouth disease in Xinjiang Uighur Autonomous Region during 2009–2018. (A) The distribution of virus subtype by age group. (B) The distribution of virus subtype by year.

In HFMD cases, 10393 (14.7%) had virus etiology tests. CV-A16 contributed the most to laboratory-confirmed cases, accounting for 39.3% of the cases. The distribution of virus subtype varied significantly by age group (*p<*0.001), that CV-A16 contributed the most in children aged 2–15 years old, and other enterovirus predominated in cases under 2 and over 15 years old (Fig 1A). Moreover, the differences in virus subtype distributions were great during the study period (*p*<0.001). Some years have one predominant virus subtype (2012, 2014–2018), others have two or more advantaged virus subtypes (Fig 1B).

**Fig 2:**
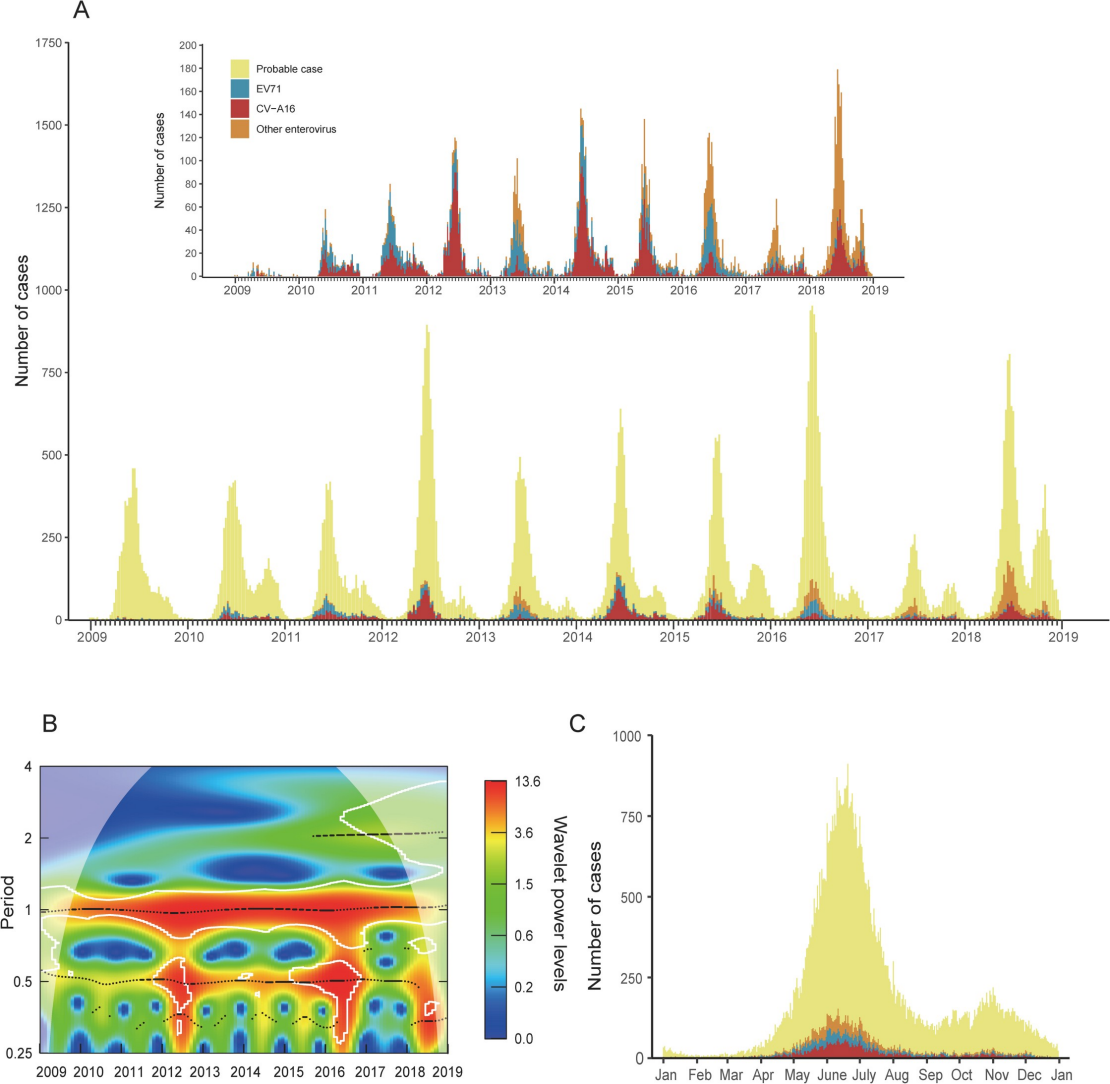
Epidemiology of hand foot and mouth disease (HFMD) in Xinjiang Uighur Autonomous Region during 2009–2018. (A) Weekly changes in HFMD incidence. (B) The periodicity of HFMD summarized by wavelet analysis. (C) Epidemical curve in the annual scale.

Weekly changes in HFMD cases incidence were summarized during 2009–2018 in Xinjiang, including probable and laboratory-confirmed cases. The incidence showed an increasing trend in 2009–2016, with two obvious peaks in 2012 and 2016. After a great decreasing of incidence in 2017, an increasing peak occurred in 2018 (Fig 2A). Wavelet power spectrum showed that the incidence of HFMD had an annual periodicity from 2009 to 2018, with apparent semi-annual periodicity in 2012, 2015, 2016 and 2018 (Fig 2B). Then, the daily incidences in Xinjiang were summarized in a year scale. Overall, the activity of disease showed semiannual peaks, including a major peak in May-July, and a minor one in October-November (Fig 2C).

**Fig 3:**
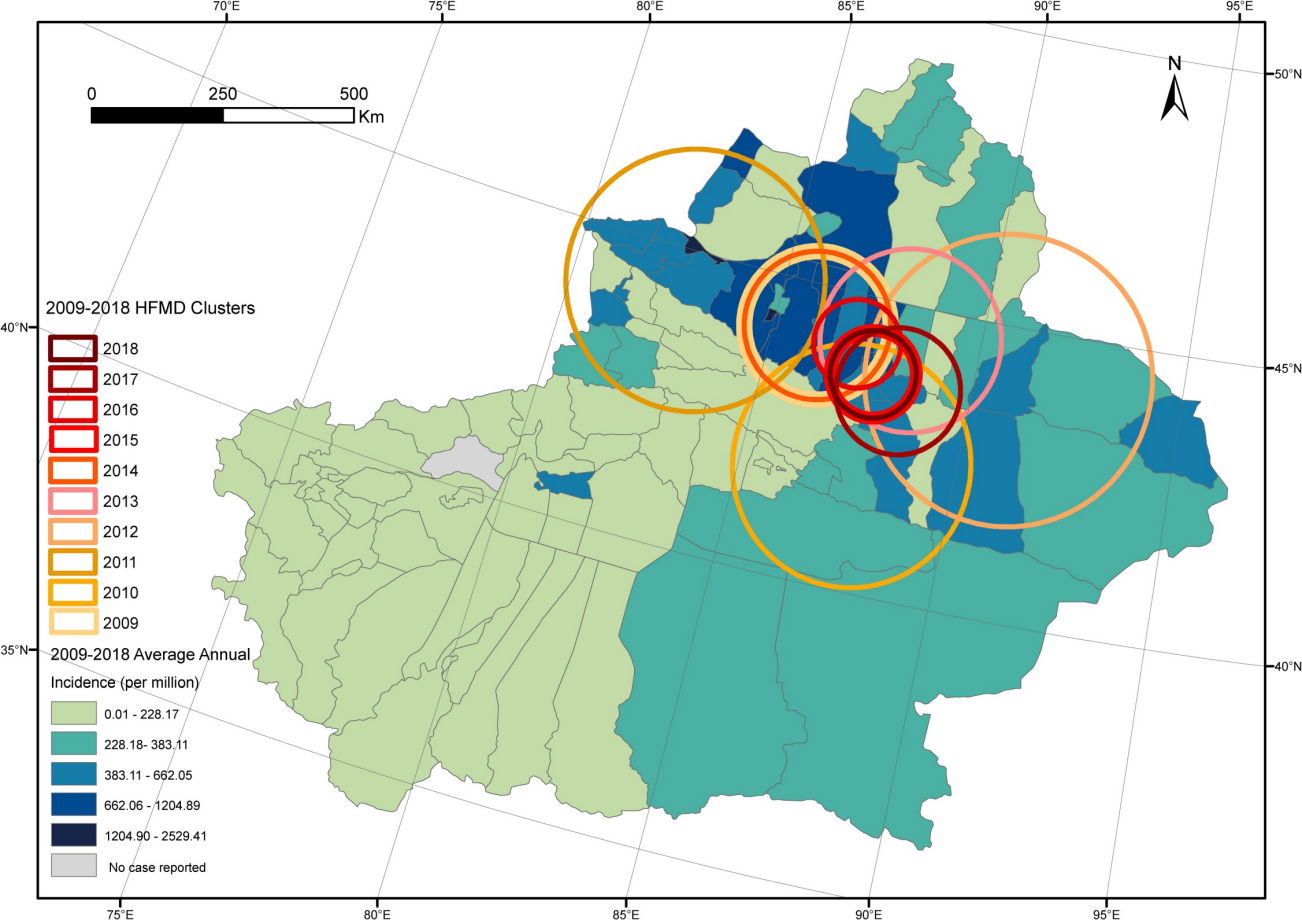
Spatial-temporal characteristics of hand foot and mouth disease (HFMD) in Xinjiang Uighur Autonomous Region during 2009–2018.

Spatial distribution pattern of HFMD in Xinjiang was mapped on county level as an average annual incidence rate (per million). Overall, the incidence rate was higher in the north than in southern area. In the north, the incidence rate was the highest where there are basins and more people living. The annual HFMD cases were clustered in 40.0°N-47.5°N from 2009 to 2018, with a radius of 82.60–275.96 km and risk ratio (RR) of 8.11–24.72. From 2009 to 2018, the affected counties were clustered around Tianshan, Dabancheng, Changji, Tianshan, Shihezi, Fukang, Muleihasake, Jinghe, Heshuo and Shihezi, respectively. Moreover, the primary clusters tended to gather over time (Fig 3).

In Xinjiang, July was the hottest season with the longest median sunshine duration (median (IQR) 98.66 (93.48, 103.84), 0.1h) and the lowest atmospheric pressure (median (IQR) 887.05 (831.29, 904.68), Hpa); January was the coldest season with smallest precipitation (median (IQR) 1.14 (0.20, 3.83), mm). In December, relative humidity (median (IQR) 63.82 (55.03, 71.79), %) and atmospheric pressure (median (IQR) 904.45 (839.22, 926.65), Hpa) were the highest; sunshine duration (median (IQR) 47.99 (39.60, 55.80), 0.1h) was the shortest (S1 Table). All demographical factors presented an increasing trend in these ten years, indicating the steady development of Xinjiang (S2 Table). The elevation in Xinjiang varied greatly from -155 m to 7573 m, with the mean value of 1492 m. In 101 counties of Xinjiang, Unused land dominated the land cover in 45 counties; 21 counties have had farm land as major type of land cover; 33 counties owned grass land as major type; two counties have water body and urban area as major land cover types, respectively (S2 Fig).

To identify the effect of climate, geographical and demographic factors on HFMD incidence, negative binomial regression was conducted. The preliminary Spearman correlation analysis indicated that the model including both monthly average sunshine duration and temperature simultaneously may suffer from collinearity (r = 0.76, *p* <0.001) (S3 Table and S4 Table), thus we constructed two separate binomial regression models. Model 1 included variables to be explored except for sunshine duration, Model 2 excluded temperature but took the sunshine duration and other covariates in. Model 1 was selected due to smaller AIC value (AIC value = 39818.51). Next, VIFs of explanatory variables in Model 1 were calculated to optimize the model, leading variables with VIF>10 removed, separately (S5 Table). Finally, the results from the model with smaller AIC value and no collinearity were presented in Table 3. From final model, we noted the urban area being major land cover type, each unit rise in monthly average temperature, monthly accumulative precipitation and GDP per capita corresponded to an increase in the risk of HFMD, with incidence risk ratios (IRRs) of 2.08 (95% CI 1.50, 2.89), 1.65 (95% CI 1.61, 1.69), 1.20 (95% CI 1.16, 1.24), and 1.14 (95% CI 1.11, 1.16), respectively. However, farmland being major land cover type, an increase of one unit in percentage of the minority and population density were related to a decreasing risk of HFMD, with the IRRs of 0.72 (95% CI 0.64, 0.81), 0.91 (95% CI 0.89, 0.93) and 0.98 (95% CI 0.98, 0.99), respectively (Table 3).

**Table 3:** Associations between climate, geographical and demographic factors and HFMD incidence ^a^.

	Univariate model		Multivariate model ^b^	
Explanatory variables (increasing unit)	IRR (95% CI)	*p*	IRR (95% CI)	*p*
Temperature (per 10°C)	1.67 (1.63, 1.71)	<0.001*	1.65 (1.61, 1.69)	<0.001*
Precipitation (per 10 mm)	1.31 (1.26, 1.35)	<0.001*	1.20 (1.16, 1.24)	<0.001*
Relative humidity (per 10%)	0.83 (0.82, 0.85)	<0.001*	Removed ^d^	
Sunshine duration (per 0.1 h)	1.03 (1.02, 1.03)	<0.001*	Removed ^c^	
Atmospheric pressure (per 10 Hpa)	0.99(0.98, 0.99)	<0.001*	Removed ^d^	
Gross domestic product per capita (per 100 000 yuan)	1.18 (1.15, 1.21)	<0.001*	1.14 (1.11, 1.16)	<0.001*
Percentage of the minority (per 10%)	0.93 (0.92, 0.95)	<0.001*	0.91 (0.89, 0.93)	<0.001*
Population density (per 100/km^2^)	0.99 (0.98, 0.99)	<0.001*	0.98 (0.98, 0.99)	<0.001*
Elevation (per 10 m)	0.999 (0.998, 0.999)	0.003*	1.000 (0.999, 1.000)	0.354
Land cover				
Unused land	Reference		Reference	
Farmland	0.75 (0.68, 0.83)	<0.001*	0.72 (0.64, 0.81)	<0.001*
Grassland	0.89 (0.81, 0.97)	0.01*	0.98 (0.89, 1.08)	0.36
Water body	1.12 (0.72, 1.74)	0.628	0.93 (0.58, 1.46)	0.66
Urban area	1.41 (1.11, 1.80)	0.005*	2.08 (1.50, 2.89)	<0.001*
School or holiday month				
School month	Not applicable		Reference	
Holiday month	Not applicable		0.70 (0.66, 0.74)	<0.001*

^a^ Incidence rate ratios (IRR) were estimated from negative binomial regression models with 95% confidence intervals (CI). School or holiday month was adjusted both in univariate and multivariate analysis, population was used as an offset.

^b^ IRR (95% CI) and *p* from Model 1 (model excluding sunshine duration).

^c^ Variable was removed because r_Spearman_ = 0.76.

^d^ Variable was removed because the VIF >10.

* *P* is statistically significant (*p* < 0.05).

## Discussion

Our study of 70856 HFMD cases reported during 2009–2018 comprehensively described the disease burden, transmission dynamics and spatial-temporal characteristics in northwestern China. The estimated average annual incidence rate (305 per million persons) is much lower than the national and coastal areas [2], which may due to the dry climate and small population density in Xinjiang. The severe illness rate (0.1%) and fatality rate (0.02%) were also very low in Xinjiang, which may be the result of low proportion of the EV71 serotype in children, as EV71 was reported to predominate in severe cases [19]. It should be noted that HFMD incidence decreased greatly in 2017 and rebounded in 2018, with the proportion of serotype EV71 decreasing constantly. The reason for this phenomenon may be the marketing of the EV71 vaccine from late 2015 in China [20]. The EV71 vaccine may have inhibited the spread of EV71, but had no significant effect on overall HFMD incidence in Xinjiang. Our age profile on HFMD agrees with reports in China and other countries that the enterovirus causing HFMD is highly transmissible in a very young age. The relatively low incidence in children under one year old may because of the antibodies from breast milk [21]. The results of our study that male (especially boys under 5 years) predominated the cases agree with conclusion of previous studies [2,5,6], and HFMD transmissibility in male was proved to be higher than female [22].

The distribution of HFMD in Xinjiang is spatial-temporal clustered mainly in summer, northern basins like Junggar Basin and Turpan Basin. This distribution pattern with annual periodicity is constant during 2009–2018. Meanwhile, semiannual periodicity was also suggested by wavelet analysis. Therefore, we summarized the data in a year scale to make the epidemic characteristics of HFMD in Xinjiang clearer. The results showed that the epidemic peak appeared in two time intervals (May-July, October-November). Previous studies showed that the major transmission rate occurring in May might be facilitated by increasing temperature and higher relative humidity [23]. the second peak of the transmission rate of childhood infectious diseases like HFMD may be associated with the school year that school opening may lead to a rise of susceptible children [24,25]. This phenomenon suggests that epidemics of HFMD in Xinjiang may also occur in autumn, the corresponding preventions should be taken as well. According to previous studies, the peaks of HFMD incidence and transmission rates are different across provinces in China, showing the necessity for describing the uniqueness of transmission rate and complex influence factors on it in Xinjiang [2,26,27].

Our study has explored potential factors on HFMD incidence. In spite of factors that have been discussed in previous studies, we included extra geographical and demographic characteristics like elevation, major land cover type, and percentage of the minority on a county scale, making the study more comprehensive. We found that higher monthly average temperature and accumulative precipitation were positively associated with the incidence of HFMD. Consistent associations have been found by studies in Singapore [28], Hong Kong [29] and mainland China [2,12,30]. Besides, other studies found the HFMD incidence was associated with relative humidity, atmospheric pressure, wind speed and sunshine duration [13,14,31]. The activity of enterovirus declining rapidly in dry environment may explain the low HFMD incidence in the season with less rainfall [32]. In addition, behavior of the host varies in different seasons, that children are more likely to play in close physical contact during summer than in winter, which will facilitate the transmission of enterovirus.

As for demographic and geographical factors, urban area as the major land cover type with higher GDP per capita was identified as the risk factor of HFMD, corresponding with the high-risk areas detected by cluster analysis in our study. Previous studies have reported inconsistent relations of GDP with HFMD incidence, without adjusting for the land using type [10,11,14]. Due to the convenient transportation and abundant educational resources, people in the urban area, especially children, have more contacts with each other for HFMD transmission. We also found protective effects of percentage of the minority, population density and farmland being major land cover type on HFMD incidence. Ang LW et al. reported consistently that HFMD incidences in Chinese (mostly the Han nationality) and Malays were significantly higher than other races in Singapore [5]. The protective effect of percentage of the minority may be related to the genetic background of different races. Previous studies reported conflicting relations of population density with HFMD incidence [14,15], without adjusting for land using type. Considering of the target population of HFMD, higher population density may not necessarily represent the higher density of children, the population density of children may even lower due to the protection from parents, reducing the risk of being infected.

Based on the risk factors we have found, multifaceted measures must be taken to prevent HFMD. The government should improve public knowledge on HFMD through intensified publicity and health education, especially in parents of under-five children. Urban area in summer and schooling period is the focus of disease prevention and control. Since it is not practical to restrain the activity of children in daily life, regularly environmental cleaning and disinfection, personal hygiene and hand washing are crucial for prevention. During the outbreak, blocking viral transmission and providing assistance to childcare organizations are major points for prevention and control of HFMD.

This study has several limitations. First, most cases of HFMD are asymptomatic and self-limiting, or some cases of HFMD do not seek for formal diagnosis and treatment, thus the case number may be underestimated by the surveillance system. Second, the epidemic characteristics of subtypes in each county were not analyzed, because each county-level CDC in Xinjiang collects the specimens of the first five mild cases and all severe cases of HFMD for etiology test every month, which may lead to bias. Third, we did not obtain the information of vaccination, which is the key factor to incidence trend and may affect our results. Fourth, our study did not estimate the potential effect of other factors, such as population mobility and doctors per 1000 persons, since we built the model on the county level and data we gathered was on larger scale. Further study may consider more on the factors above and HFMD.

In conclusion, our study found the constant lower incidence, severity, and mortality in Xinjiang compared to the national level during 2009–2018. A large number of climate, geographical and demographic characteristics including temperature, precipitation, GDP per capita, population density, schooling, percentage of the minority and land cover type were found to be related to HFMD incidence. Further studies are warranted to explore the HFMD epidemic characteristics and risk factors to strengthen the intervention measures.

## Supporting information

S1 Text Panel negative binomial regression for the effects of multiple factors on hand foot and mouth disease (HFMD). (DOCX)

S1 Fig Geographical location of Xinjiang Uighur Autonomous Region. (TIF)

S2 Fig Elevation and types of land cover in Xinjiang Uighur Autonomous Region. (TIF)

S1 Table Monthly distributions (median (IQR)) of meteorological factors in Xinjiang counties from 2009 to 2018. (XLSX)

S2 Table Annual distributions (median (IQR)) of demographical factors in Xinjiang counties from 2009 to 2018. (XLSX)

S3 Table Spearman correlation matrix of meteorological, geographical and demographic variables. (XLSX)

S4 Table P-values for Spearman correlation of meteorological, geographical and demographic variables. (XLSX)

S5 Table Variance inflation factors (VIFs) of variables in multivariate models. (XLSX)
