# Supplementary material for: Spatial-temporal mapping and risk factors for hand foot and mouth disease in northwestern inland China
Source: PLoS Negl Trop Dis. 2021 Mar 24;15(3):e0009210. doi: 10.1371/journal.pntd.0009210 (PMC8021183; doi:10.1371/journal.pntd.0009210)
Supplement: S1 Text — (DOCX) [file pntd.0009210.s001.docx]

**S1 Text. Panel negative binomial regression for the effects of multiple factors on hand foot and mouth disease (HFMD).**

We aimed to describe the correlation between the intensity of HFMD epidemiology and climate, geographical and demographic factors in order to identify good predictors of high risk.

S1.1 Data and data exploration

The intensity of local transmission of HFMD was estimated by counting the number of cases per county *i* in every month *t* from 2009 to 2018. The number of cases was selected to be dependent variable in panel negative binomial regression model.

Climate variables included: monthly mean temperature (T, ℃), relative humidity (RH, %), atmospheric pressure (AP, Hpa) and sunshine duration (SSD, 0.1h), monthly accumulative precipitation (PRE, mm). Monthly distributions of meteorological factors in Xinjiang counties were presented in S1 Table. July was the hottest season with the longest median SSD (median (IQR) 98.66 (93.48, 103.84)) and the lowest AP (median (IQR) 887.05 (831.29, 904.68)); January was the coldest season with smallest PRE (median (IQR) 1.14 (0.20, 3.83)). In December, RH (median (IQR) 63.82 (55.03, 71.79)) and AP (median (IQR) 904.45 (839.22, 926.65)) were the highest; SSD (median (IQR) 47.99 (39.60, 55.80)) was the shortest (S1 Table).

Demographical factors included: the yearly gross domestic product per capita (GDP per capita, 10 000 yuan), percentage of the minority (PM, %), and population density (PD, per /km^2^). Annual distributions of demographical factors in Xinjiang counties from 2009 to 2018 were presented in S2 Table. All demographical factors presented an increasing trend in these ten years, indicating the steady development of Xinjiang (S2 Table).

Geographical variables included: mean elevation (ELE, m), major type of land cover (LC, farmland, forest, grassland, water body, urban area and unused land). The elevation in Xinjiang varied greatly from -155 m to 7573 m, with the mean value of 1492 m. In 101 counties of Xinjiang, Unused land dominated the land cover in 45 counties; 21 counties have had farm land as major type of land cover; 33 counties owned grass land as major type; two counties have water body and urban area as major land cover types, respectively (S2 Fig).

To control the potential confounds caused by schooling of students, January, February, July and August were classified as holiday months according to the Chinese system of winter and summer holidays arrangement. Other months were defined as school month. School or holiday month was included into both the univariate model and multivariate model.

Collinearity between factors to be included into the model was expected. Spearman correlation coefficient was calculated between pairwise continuous factors, and the correlation matrix was presented (S3 Table and S4 Table).

S1.2 Panel negative binomial regression model

S1.2.1 Model description

We used fixed-effects panel negative binomial regression model in our study, which is an overdispersion models. Before we conducted the model, panel setting was conducted declaring our data to be a panel data. Each county was set as a panel, in which the order of observations is relevant, thus controlling the time-trend. We let y*_it_* be the count of cases for the *t*th month in the *i*th county (group) of Xinjiang. We begin with the model *y_it_|γ_it_* ~ Poisson(γ*_it_*), where *γ_it_|δ_i_* ~ gamma(*λ_it_,δ_i_*) with *λ_it_*=exp(*x_it_β*+offset*_it_*) and *δ_i_* is the dispersion parameter. This yields the model

Pr ($Y_{it}=y_{it}|x_{it},\delta_{i}$) $=$ ${\frac{\Gamma(\lambda_{it}+y_{it})}{\Gamma(\lambda_{it})\Gamma(y_{it}+1)}\left( \frac{1}{1+\delta_{i}} \right)}^{\lambda_{it}}\left( \frac{\delta_{i}}{1+\delta_{i}} \right)^{y_{it}}$

(See Hausman JA, Bronwyn H and Zvi G [1]; our *δ* is the inverse of their *δ*.) Looking at within-panel effects only, specification yields a negative binomial model for the *i*th group with dispersion (variance divided by the mean) equal to 1+*δ_i_*, that is, constant dispersion within group.

For the fixed-effects overdispersion model, we condition the joint probability of the counts for each group on the sum of the counts for the group (that is, the observed $\sum_{t=1}^{n_{i}} y_{it}$). This yields

Pr ($Y_{i1}=y_{i1},\ldots,Y_{{in}_{i}}=y_{{in}_{i}}|X_{i},\sum_{t=1}^{n_{i}} Y_{it}=\sum_{t=1}^{n_{i}} y_{it}$)

$$= \frac{\Gamma(\sum_{t=1}^{n_{i}} \lambda_{it})\Gamma(\sum_{t=1}^{n_{i}} y_{it}+1)}{\Gamma(\sum_{t=1}^{n_{i}} \lambda_{it}+\sum_{t=1}^{n_{i}} y_{it})}\prod_{t=1}^{n_{i}} \frac{\Gamma(\lambda_{it}+y_{it})}{\Gamma(\lambda_{it})\Gamma(y_{it}+1)}$$

The conditional log likelihood is

$$\ln L=\sum_{i=1}^{n} \omega_{i}[\ln\Gamma\left( \sum_{t=1}^{n_{i}} \lambda_{it} \right)+\ln\Gamma\left( \sum_{t=1}^{n_{i}} y_{it}+1 \right)-\ln\Gamma\left( \sum_{t=1}^{n_{i}} \lambda_{it}+\sum_{t=1}^{n_{i}} y_{it} \right)+\sum_{t=1}^{n_{i}} \{\ln\Gamma\left( \lambda_{it}+y_{it} \right)-\ln\Gamma\left( \lambda_{it} \right)-\ln\Gamma\left( y_{it}+1 \right)\}]$$

We set the penal using *xtset* commands, and conducted fixed-effects panel negative binomial regression model using *xtnbreg* commands in Stata15.1.

S1.2.2 Model selection

Because a pair of meteorological variables were highly correlated (T and SSD), and including correlated factors in the model may result in misleading, we used a strategy using Akaike information criterion (AIC) as selection criteria. We started with two possible multivariate models (Model 1 and Model 2) with one of correlated variables and other significant (p<0.1) variables in univariate model, and chose the one with smaller AIC. In addition, variance inflation factors (VIF) were also calculated to avoid collinearity of the selected model, explanatory variables with VIF>10 were removed from the model step by step.

S1.2.3 Results of model selection

In the univariate models, all variables were significant to be included into multivariate model (Table 3). Therefore, two multivariate models were built. Model 1 included T with other significant variables in univariate models but SSD, and the AIC value was 39818.51; Model 2 included SSD with other variables but T, the AIC value was 40784.42. So, Model 1 was selected due to lower AIC. Then, we calculated VIFs of each variable in Model 1, and the VIF of RH was over 10 being the largest valued 34.24. Therefore, we removed RH from Model 1 and repeat panel negative binomial regression, thus built the Model 3. The VIFs of variables in Model 3 indicated that including AP (VIF=13.17) caused the collinearity, thus we removed AP from Model 3 and the remaining variables constituted Model 4. Model 4 was the final model, including T, PRE, GDP, PM, PD, ELE and LC with school or holiday month adjusted, the VIFs of explanatory variables in Model 4 were all less than 10 (S5 Table).

[1] Hausman JA, Bronwyn H, Zvi G. Econometric Models for Count Data with an Application to the Patents-R&D Relationship (October 1984). NBER Working Paper No. t0017, Available at SSRN: https://ssrn.com/abstract=284047.
