# Supplementary figures and images for: Spatial-temporal mapping and risk factors for hand foot and mouth disease in northwestern inland China
Source: PLoS Negl Trop Dis. 2021 Mar 24;15(3):e0009210. doi: 10.1371/journal.pntd.0009210 (PMC8021183; doi:10.1371/journal.pntd.0009210)

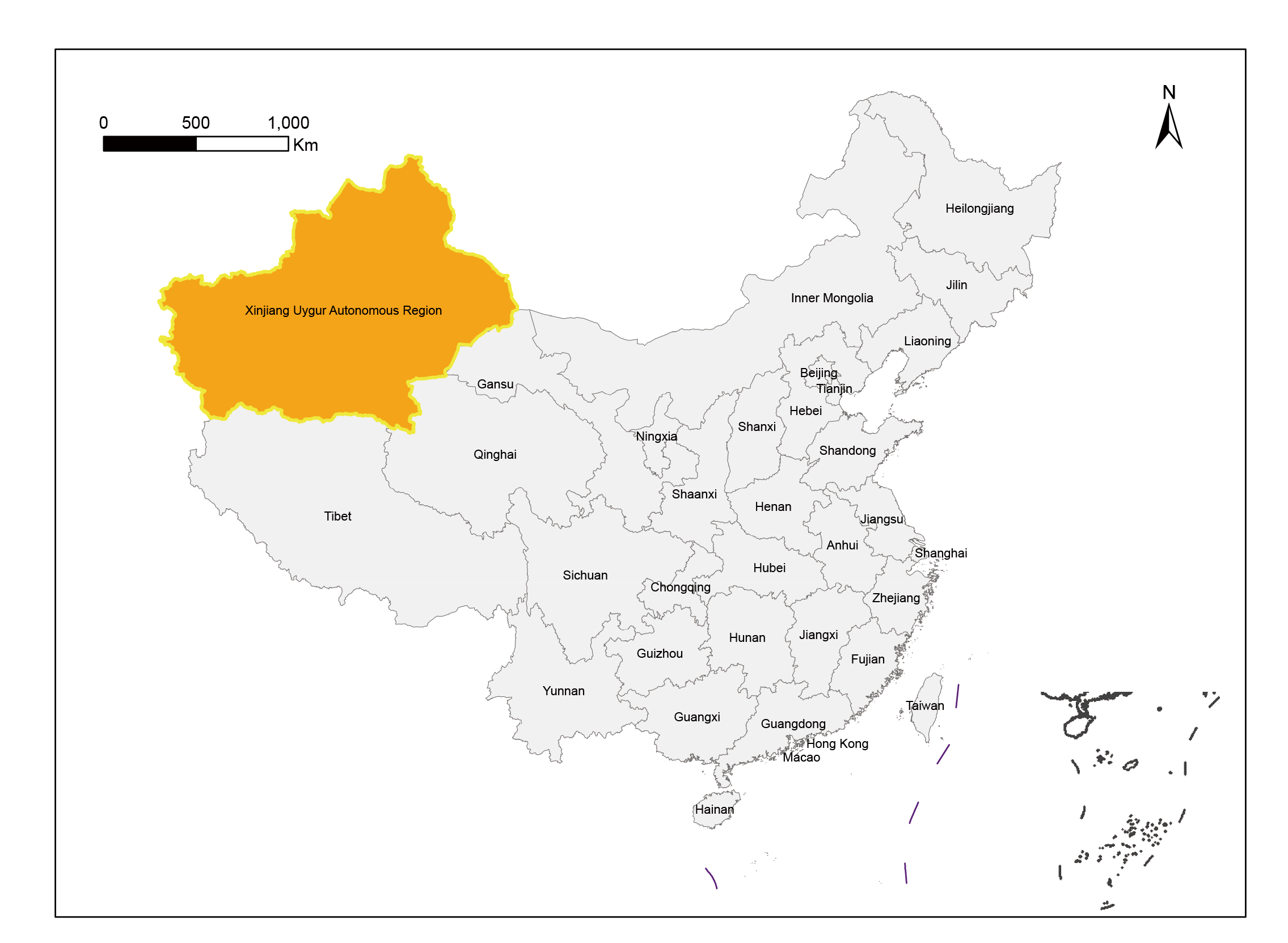

Supplement: S1 Fig — (TIF) [file pntd.0009210.s002.tif]

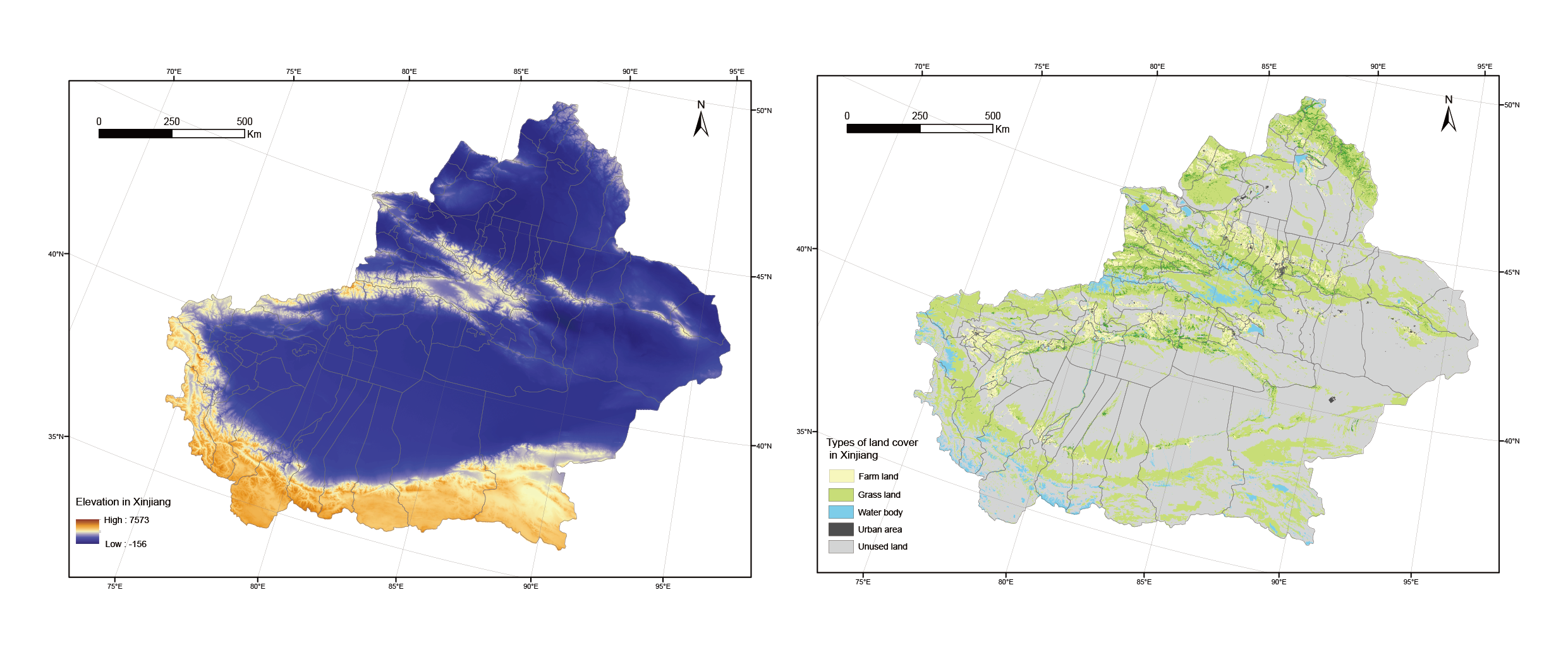

Supplement: S2 Fig — (TIF) [file pntd.0009210.s003.tif]
